# Case of Multiple Abscess

**Published:** 1886-11-20

**Authors:** J. McF. Gaston

**Affiliations:** Professor of the Principles and Practice of Surgery


### CASE OF MULTIPLE ABSCESS.

*A Clinical Lecture at the Southern Medical College, Atlanta, Ga.*

BY J. McF. GASTON, M. D.,
Professor of the Principles and Practice of Surgery.

REPORTED FOR THE SOUTHERN MEDICAL RECORD.

We present to the Class to-day a striking exemplification of the influence of the lowering of vital force as a predisposing cause of inflammation, which has been considered in the regular lectures this morning. No operative measure is indicated at present in this patient; but it is by no means the most instructive case for the student which calls for a surgical operation. An essential phase of Surgery is to cure when possible without the knife, and yet I will have occasion to report in the history of the previous treatment of this disease various operative measures.

This colored man—about thirty years of age—enjoyed complete health until attacked with typhoid pneumonia some twenty months ago; after which he labored under a general prostration, terminating in the development of several tumors in the lumbar and femoral regions. He entered the Ivy Street Hospital on the first day of August of the current year.

Upon examination I found a large tumor extending from the left lumbar across to the hypochrondriac region of the same side and reaching below the points of the false ribs, in which fluctuation was evident, but rather deep seated. There was no indication of pointing, or thinning of the superficial tissues, but a general protuberance. An enlargement of the structures composing the posterior aspect of the left thigh immediately below the gluteal region, with evidence of fluctuation in a limited area, was also very perceptible, and materially interfered with locomotion. There were two other prominences near the sacrum, one of which could be traced towards the left iliac region.

It was thought proper to make an exploratory puncture at that time in the left lumbar region; and finding more than usual resistance after passing through the subcutaneous cellular tissue, the instrument was thrust into a cavity, from which pus flowed through the canula, of a character corresponding to that observed in scrofulous abscesses. With the recognized practice of avoiding a free opening in such cases, I employed the aspirator for drawing off the contents, but found that all the accumulation was not discharged, owing to the clogging of the tube. On the day following I

concluded to take the choice of evils, and make a free incision into the abscess, when again I was impressed with the dense nature of the investing tissue, and considerable force was required to penetrate the cavity with a sharp-pointed bistoury. A copious discharge of purulent fluid, mixed with cheesy flocculi, resulted from this free opening, and a tube of eight inches in length was left in the cavity to effect thorough drainage, while a firm compress was secured by a bandage around the body.

I wish to impress upon you the great importance, in these extensive abscesses, of approximating the walls by compression at all points except immediately around the external opening, so as to agglutinate the tissues of the pyogenic membrane. If all the contents of an abscess are evacuated, a species of vacuum is left, and there is great proneness to redevelopment, unless such compression is resorted to and kept up continuously, to form the union by granulations between the approximated walls.

The presence of a pyogenic membrane in all abscesses serves to protect the adjacent tissues from contact with the pus, and without this provision purulent absorption would doubtless occur in most instances of suppuration ; but the dense and resisting nature of the pyogenic membrane was a remarkable feature of this case. It was found that the discharge diminished in a few days, so as to render the drainage tube unnecessary, and, with the application of absorbent cotton over the opening, and some folds of cloth as a compress, the cavity was obliterated within a week, so that there was only a slight collection immediately around the opening made by the knife.

My attention was next directed to the abscess of the thigh, which was treated in the same manner as detailed in the lumbar abscess; but, owing to the permeation of the fascia and intermuscular spaces by the purulent collection, the result has not been so favorable. There is still considerable induration of the deep structure, as I perceive upon palpation ; and you observe the original opening has not closed. As proper compression around the entire limb could not be made without impeding the circulation, it has not been practicable to effect such approximation of the walls of the pyognic membrane as to agglutinate them.

You observe upon the dorsal surface the indications of these other openings of distinct abscesses, but of much less extent than the two which have been pointed out ; and at present there are no new developments of a purulent nature.

As this condition is attributable to the altered state of the secretory and absorbent apparatus along with impaired nutrition, it has been my aim throughout to restore tone to the organization and correct this deteriorated condition of the blood elements. He was given Lugol's Solution of Iodine, and iodide of potash, continuously, with the extract of malt, during the first month. This was followed by the syrup of iodide of iron and cod-liver oil, with the most nutritious food ; but finding that an œdematous condition of the extremities, with puffing of all parts of the body, ensued, he was ordered $\frac{1}{4}$ grain of the proto-iodide of mercury three times a day, and, with the continuation of this for ten days, you perceive there is no longer any œdema, while his general appearance has materially improved.

With the therapeutic agency of this alterative, and the future resort to tonics, it is expected that there will be no further purulent developments.

The immediate or exciting cause of multiple abscesses, such as we have in this patient, is doubtless embolism—since they are not the result of any local irritant, nor can they be attributed to micrococci. In the pus from cold abscesses, Rosenthal was unable to find any micro-organisms ; and, although there is much in favor of the idea that suppuration is dependent upon the presence of micro-organisms, yet it cannot, at the present time, according to the Reference Hand-book, be stated as absolutely proven.

In the adynamic state of the vaso-motor system of nerves connected with the atony of the circulatory apparatus, both central and peripheral, attending the general prostration under which this man labored for more than a year, it is a fair inference that some thromboid vessels existed, which originated this trouble, accompanied with scrofulous cachexy.

We have now relieved the local effects by the evacuation of five different collections of pus, but the more difficult task of correcting the wide-spread deterioration in the nutritive functions is yet to be accomplished. The disorder is not local, but constitutional, and must be treated accordingly by remedies calculated to recuperate the vital forces.

